# Hypertriglyceridemia—Causes, Significance, and Approaches to Therapy

**DOI:** 10.3389/fendo.2020.00616

**Published:** 2020-09-02

**Authors:** Leinys S. Santos-Baez, Henry N. Ginsberg

**Affiliations:** Department of Medicine, Division of Preventive Medicine and Nutrition, Vagelos College of Physicians and Surgeons, Columbia University, New York, NY, United States

**Keywords:** hypertriglyceridemia, triglycerides, lipids, lipoproteins, chylomicrons, very low density lipoproteins, apolipoproteinB, novel therapeutics

## Abstract

Hypertriglyceridemia (HTG) is a common metabolic disorder with both genetic and lifestyle factors playing significant roles in its pathophysiology. HTG poses a risk for the development of cardiovascular disease (CVD) in the population at large and for pancreatitis in about two percent of individuals with extremely high levels of triglycerides (TG). This manuscript summarizes the mechanisms underlying the development of HTG as well as its management, including emerging therapies targeted at specific molecular pathways.

## Introduction

Triglycerides (TG) are an essential molecule for efficient storage of excess energy in the body. Whenever we have excess nutrients, we store them as fat, which is TG; this is true whether the excess nutrient is carbohydrate, protein, or fat itself. TG travels through the blood stream on lipoproteins; about 90% circulates with chylomicrons and very low-density lipoproteins (VLDL). Chylomicrons (CM) carry dietary fats that were metabolized to fatty acids (FA) and mono- or diglycerides in the small intestine (SI) before being absorbed by enterocytes and reassembled into TG. The latter, along with cholesterol, phospholipids, and apolipoprotein B48 (apoB48) are assembled into CM and secreted into the lymphatic system and then into the circulation where they can deliver their TG-FA to adipose tissue for storage or to both skeletal and cardiac muscle to be used for energy. What remains at this stage is called the CM remnant (R) that carries dietary cholesterol, along with cholesterol transferred to CM from high density lipoproteins (HDL) and low density lipoproteins (LDL) via cholesterol ester transfer protein (CETP). TG is synthesized in the liver from adipose tissue-derived FAs, from FA generated within the liver from acetylCoA (*de novo* lipogenesis or DNL), and from FA released from CM-R and VLDL-R TG that have escaped peripheral uptake resulting in removal from the circulation by the liver (1, 2). Hepatic TG can be stored as cytosolic lipid droplets, oxidized in mitochondria, or assembled in to VLDL along with cholesterol, phospholipids, and apolipoprotein B100 (apoB100).

Based on population studies, about one-quarter of the population has a fasting plasma TG level below 100 mg/dl—a level considered as very normal. Approximately 65% of people have levels <150 mg/dl, a value often considered as the upper limit of normal. However, the full range of fasting plasma TG is from about 30 mg/dl to 10,000 mg/dl (0.33–12 mmol/L) (3). Importantly, the risk for cardiovascular disease (CVD) increases across the range of TG, at least until it is about 1,000 mg/dl, at which point the major risk becomes acute pancreatitis, although risk for CVD remains increased. In this review, we will examine why plasma TG levels can vary so widely and why moderate to very high levels can be associated with risk for CVD and pancreatitis. Finally, we will discuss existing as well as future therapies that may reduce or eliminate the risk for both of these potentially life-altering outcomes.

## Regulation of Plasma TG Levels

A detailed description of the assembly and secretion of CMs and VLDL is beyond the scope of this review (4). Key points, based on studies in cells, mice, and people with normal and elevated levels of plasma TG include the following: In both the SI and liver, assembly and secretion of apoB48 in CM and apoB100 in VLDL, respectively, are regulated mainly at the post-transcriptional and post-translational stages. Thus, in both the SI and the liver, transcription of the APOB gene is, with few exceptions, constitutive, as is translation of its mRNA. This is consistent with a model in which apoB48, with a molecular mass of over 200 kD, and apoB100, with a molecular mass of over 500 kD, must be available for sudden increases in the need to TG transport out of the SI and liver, respectively, during periods of fluctuating arrival of FAs. In turn, the constant synthesis of apoB in each organ must be balanced by secretion of CMs or VLDL, or degradation of apoB, which has been shown to occur as early as co-translationally and as late as post-endoplasmic reticulum. In the SI, the rapid influx of large quantities of FA after ingestion of dietary fat resulting in high rates of TG synthesis, must be balanced by assembly and secretion of CMs. In the liver, as noted above, the pool of TG derives from multiple sources and it is not clear which (DNL, FA delivery from the circulation, or FA released from lysosomes after degradation of CM-R and VLDL-R) is the dominant stimulus for VLDL secretion. A series of TG-synthetic enzymes, culminating in the diacylglycerol acyltransferases, DGAT1 and DGAT2, and microsomal triglyceride transfer protein (MTP) play key roles, but the rate of cholesterol synthesis and size of the hepatic cholesterol pool are also factors in the secretion of VLDL (1).

In summary, both CMs and VLDL are carriers of energy, in the form of TG, from the SI and the liver to adipose tissue and muscle for storage or utilization, respectively. Disorders of nutrient balance, whether due to excess caloric intake or inefficient storage of energy, will result, therefore, in increased rates of secretion of TG from the SI and liver. Since obesity and insulin resistance are common throughout the world, moderate HTG is nearly always associated with increased rates of CM and VLDL secretion (1, 5). However, two studies using tracer kinetic methods, published 50 years apart (6, 7), remind us that fractional clearance of TG from circulating chylomicrons and VLDL is also a critical determinant of plasma TG concentrations. It is crucial to understand that under steady state conditions, the concentration of any circulating molecular species is dependent on both the absolute rate of its entry into the circulation, e.g., secretion rate, and the efficiency with which it is removed, e.g., fractional clearance rate. At steady state, absolute secretion rates and absolute clearance rates are equal—it is the efficiency of clearance, or fractional clearance rate, which determines the concentration of a molecule for any level of its absolute secretion rate.

A simplified model of the clearance of CMs and VLDL comprises a two-step process: Lipolysis of circulating TG in both by lipoprotein lipase (LpL) followed by hepatic clearance of CM-R and VLDL-R [the latter are often called intermediate density lipoprotein (IDL) particles], respectively. For several decades, TG lipolysis by LpL appeared to be a relatively simple process, with binding of the TG-rich lipoproteins to LpL on the surface of capillary endothelial cells in adipose or muscle tissues, with apolipoprotein CII (apoCII), the necessary activator of LpL, and apolipoprotein C III (apoCIII), an inhibitor of lipolysis, playing key regulatory roles. In the past two decades, the story has become much more complex with the discovery of apoA5, angiopoietin-like protein 3 (angptl3), angiopoietin-like protein 4 (angptl4), lipase maturation protein (LMF), and glycosylphosphatidylinositol anchored high density lipoprotein binding protein (GPIHBP1). A full description of their roles is beyond this discussion other than to note that LOF mutations in the genes for these proteins can cause, particularly in the homozygous state, severe HTG (LpL, apoCII, apoA5, LMF, and GPIHBP1) or hypolipidemia (apoCIII, angptl3, and angptl4) (8, 9). Heterozygosity for TG raising mutations can also play a role in polygenic HTG (3).

All CM-Rs and variable amounts of VLDL-Rs are cleared by the liver. Importantly, VLDL-Rs can undergo further loss of TG via LpL and hepatic lipase (HL) mediated lipolysis, to become LDL. Hepatic clearance of remnants occurs mainly via the LDL receptor, with an important role for the LDL receptor-related protein-1 (LRP1) and heparin sulfate proteoglycans, particularly syndican1. For CM-Rs, hepatic clearance requires functional apoE, as apoB48 is not a ligand for LDL receptors. In contrast, VLDL-Rs can use either apoB100 or apoE. ApoE has three isoforms that differ by whether they have a cysteine or arginine at amino acids 112 and 158. ApoE 2 has cysteines at both sites and binds very poorly to both the LDL receptor and LRP-1; apoE3 has a cysteine at amino acid 112 and an arginine at 158 whereas apoE4 has arginines at both sites. ApoE3 (by far the most abundant isoform) and apoE4 bind with high affinity to both receptors. The relative roles of apoB100 and apoE in remnant uptake by the liver depends on a number of factors and cannot be definitively quantified. Importantly, apoCIII inhibits remnant uptake by the liver (10). The proportion of VLDL that is removed directly as a remnant vs. that which is converted to LDL ranges from 25 to 75%; regulation of the path that a VLDL particle takes is not understood completely, although LpL activity appears to play a critical role (10, 11).

## Hypertriglyceridemia

As noted above, the full range of fasting plasma TG is from about 30 mg/dl to 10,000 mg/dl (0.33–12 mmol/L) (3). Levels above 150 mg/dl (1.7 mmol/L) are found in about 30% of the population. Severe HTG, defined as plasma TG concentration >10 mmol/L (>885 mg/dL), is uncommon, with a prevalence ranging from 0.10 to 0.20%, while very severe HTG, defined at TG >20 mmol/L (>1,800 mg/dL) is rarer still (prevalence 0.01%). Quantifying the abnormal lipoprotein species in patients with elevated TG, as seen in the Fredrickson or WHO classification of hyperlipidemias, is technically complicated (12). For this reason, the general term “HTG” is usually sufficient for clinical purposes. Primary severe HTG has both monogenic and polygenic determinants. A tiny subset of these patients (perhaps 2% of individual with severe HTG) have monogenic chylomicronemia or Familial Chylomicronemia Syndrome (FCS, former type 1), a rare form of monogenic HTG with estimated prevalence of 1 to 10 in a million people. Definitive diagnosis of this autosomal recessive disorder requires molecular detection of rare, biallelic (homozygous or compound heterozygous) variants in one of five genes: *LPL* (accounting for 80% of cases), *APOC2, APOA5, LMF1*, and *GPIHBP1*. The roles of these gene products in lipolysis have been defined in several publications and will not be included here (9, 13–15). Genetic assessment has superseded biochemical assays of plasma lipolytic activity as the gold standard for diagnosis.

Most remaining cases of severe HTG are polygenic in nature, which includes contributions from rare heterozygous variants in the above five canonical FCS genes and/or accumulated common variants associated with elevated TG levels identified in genome-wide association studies (3). Sometimes referred to as multifactorial HTG (former type 5), certain secondary factors, such as diabetes mellitus, obesity, estrogen treatment, alcohol and high fat diets, can further exacerbate the effects of genes on severely elevated TG levels.

## Triglycerides as a Risk Factor for Pancreatitis

The role of HTG in the etiology of pancreatitis has been reviewed recently and will only be summarized here (16, 17). It is clear that severe HTG may lead to acute pancreatitis and that overall, HTG is causative in a significant proportion of acute pancreatitis events. It is, however, unclear whether all TRLs carry the same risk or whether CMs and CM-R carry a greater risk than VLDL and VLDL-R. Since a mixture of TRLs is present in the majority of severe HTG because of the competition of VLDL and CM for LpL, it is difficult to assign a specific TRL to the onset of pancreatitis. Understanding the pathophysiologic link between HTG and pancreatitis has been confounded by the absence of a clear cutpoint in TG concentrations and risk. In fact, the majority of subjects with TG >10 mmol/L (885 mg/dL) will never experience pancreatitis. However, the rare patients with monogenic chylomicronemia with low to undetectable levels of remnants and downstream lipoproteins due to severely impaired lipolytic machinery, have the greatest risk for pancreatitis (16). How very high TG concentrations cause pancreatitis is still unclear, although the idea that local, very high levels of FAs liberated from TG by pancreatic lipase within the pancreas sets off a cycle of damage, more liberation of lipase, more lipolysis locally, and more damage (18, 19).

## Triglycerides as a Risk Factor for CVD

The role of HTG in CVD has been a controversial subject for decades (2, 16, 20–23). The basis for the controversy is the complexity of lipoprotein-lipid metabolism and the shortcomings of population-based epidemiologic studies where multivariate analyses cannot always adequately dissect physiologic interactions adequately. TG, because of its role in energy metabolism, has a much greater day-to-day variability in blood levels than do LDL and HDL cholesterol, weakening its statistical power. More importantly, VLDL TG levels are, via the action of CETP, inversely related to HDL cholesterol levels. Possibly most importantly, VLDL carries TG and a significant quantity of cholesterol, rendering adjustment of the role of TG in CVD risk by VLDL cholesterol (which is typically calculated in part from TG concentrations) of questionable value (20). More recently, examination of variants in genes involved in TG metabolism and HTG have strengthened significantly the argument that TG, or at least TG-rich lipoproteins, are causally linked to CVD (24–27).

## Therapeutic Approaches for Hypertriglyceridemia

### Lifestyle

The initial and most valuable approach is an adjustment in lifestyle contributors to HTG. These include management of diet, weight, and the non-TG components of the metabolic syndrome. There are medications that can elevate TG levels, including corticosteroids, thiazides, nonselective beta-blockers, estrogen, tamoxifen, bile acid sequestrants, cyclophosphamide, antiretroviral drugs, and second-generation antipsychotic agents. Other contributors to HTG that should be examined include renal disease, hypothyroidism, diabetes mellitus, pregnancy, paraproteinemia, and some auto-immune diseases (2, 16, 28). Unfortunately, lifestyle approaches have proven unreliable since they depend on patient adherence (2, 16, 28).

### Fibrates

Fibrates, which are generally considered peroxisome proliferator-activated receptor alpha (PPARα) agonists, have been used to treat HTG for more than 40 years. Despite excellent efficacy as TG-lowering drugs, their effects on CVD were, however, inconsistent in monotherapy trials. In the only trial of a fibrate in combination with statin therapy (the ACCORD Trial), there was no significant benefit overall, although a pre-specified subgroup analysis of participants with TG in the upper tertile (>204 mg/dl) and HDL cholesterol in the lower tertile (<35 mg/dl) found a 28% reduction in major adverse cardiac events, (MACE) compared to placebo (29).

Pemafibrate (K-877; developed by Kowa Pharaceuticals), the first fibrate to be developed since ACCORD, is a selective peroxisome proliferator-activated receptor-α modulator (SPPARMα) (30). In a systematic review of studies performed in Japan, pemafibrate reduced plasma triglyceride (TG) levels and increased high-density lipoprotein cholesterol (HDL-C) with efficacy similar to fenofibrate, and had a good safety profile (31). Unlike fenofibrate, that increases serum creatinine, there was minimal effect of pemafibrate on creatinine levels. Pemafibrate's ability to reduce CVD is being evaluated in the PROMINENT study (Pemafibrate to Reduce Cardiovascular Outcomes by Reducing Triglycerides in Patients with Diabetes), a phase 3 randomized clinical trial with an estimated enrollment of 10,000 participants with plasma TG and HDL levels similar to those of the dyslipidemic subgroup in ACCORD. This trial is will be completed in 2022 (32).

### Omega-3 Fatty Acids

Omega 3 fatty acids are polyunsaturated FAs (PUFAs) that have a range of important roles in cell physiology (33). They are essential fatty acids because they cannot be synthesized *de novo* from small carbon molecules in the body. The main omega 3 FAs are alpha-linolenic acid (ALA), which can be obtained from plant oils, including flaxseed, soybean, and canola, and both eicosapentaenoic acid (EPA) and docosahexaenoic acid (DHA), which must be obtained from consuming fish and other seafood, although small quantities can be generated by elongation of ALA. Twenty-one years ago, the GISSI study of 1 gm/day supplementation with a combination of DHA/EPA ethyl esters in the ration of 2:1 demonstrated a significant protective effect in patients with a recent myocardial infarction (33, 34). However, studies that followed were, for the most part negative (35), including two recent primary prevention studies, ASCEND and VITAL. In these two studies, more than 15,000 and 25,000 individuals, respectively, were randomized to one gm/day of DHA/EPA or placebo and followed for a mean of 7.4 and 5.3 years, respectively (36, 37). Of note, the JELIS study of 1,800 mg EPA/day plus statin vs. statin alone in 18,645 Japanese patients followed for a mean of 4.6 years demonstrated a significant 19% benefit on major coronary events (38). This study, however, was randomized but not blinded, and this led to concerns about the results.

REDUCE–IT (Reduction of Cardiovascular Events With EPA - Intervention Trial), was a randomized double-blind study that evaluated the effects of icosapent ethyl, a purified EPA ethyl ester (developed by Amarin), in subjects taking statins with plasma TG levels of 150–499 mg/dl. The subjects had either a prior cardiovascular event or diabetes mellitus with additional risk factors. The median follow-up was 4.9 years. Compared to the placebo group, subjects who received 4 gms/day of icosapent ethyl had a 25% reduction in the primary endpoint of MACE, with a number needed to treat of 21. Of note, although placebo-corrected reductions in TG of 19% were achieved by icosapent ethyl, there was no relationship between baseline TG and the benefit on CVD. The results of REDUCE-IT, together with those from JELIS, suggest that EPA has uniquely beneficial effects on CVD (39). In contrast, a very recent expanded meta-regression of 13 trials of omega-3 FAs that included ASCEND, VITAL, and JELLIS, but not REDUCE-IT, and had over 127,000 participants, found significant beneficial effects of omega-3 FAs, mainly with DHA:EPA combinations (40).

### Treatments to Reduce ApoCIII

ApoCIII is synthesized in hepatocytes and in the intestine. It is carried in the circulation mainly on VLDL and CM, but also on HDL and to a lesser extent, LDL. As noted earlier in this review, apoCIII inhibits the activity of LPL as well as the uptake of remnants by the liver (10, 41). LOF mutations in *APOC3* have been associated with reduced TG levels and a reduced risk of CVD (24, 25, 42). Volanesorsen, an antisense oligonucleotide (ASO) (developed by Ionis Pharmaceuticals, Inc.) targeted against apoC-III mRNA, was developed for the treatment of FCS, severe HTG, and familial partial lipodystrophy (FPL) (43). The US Food and Drug Administration did not approve volanesorsen, but the European Medicines Agency did, in 2019, basing this decision on the positive results of Phase 2 and 3 trials. A key study was the APPROACH trial, a double blind phase 3 trial that evaluated the efficacy and safety of volanesorsen in adults diagnosed with FCS, who at the time of enrollment, were unsuccessfully treated with a restrictive diet, fibrates, statins or other lipid lowering agents (44). Sixty-six subjects enrolled: 41 were homozygous or heterozygous for *LpL* LOF mutations and 11 were either double heterozygotes for LOF mutations in LpL and another accessory protein (*ApoA5, LMF1*) or biallelic for mutations in *ApoA5, LMF, GPIHBP1*, or *ApoC2*. They were randomized to placebo or 300 mg volanesorsen weekly for 52 weeks. At 3 months, TG levels were reduced by 77% (1,712 mg/dl) and this reduction was maintained for the duration of the study. Adverse events included skin reactions in 61% of subjects and platelet counts falling to <100,000 in nearly 50% of subjects taking volanesorsen. The ability of apoCIII inhibition to reduced plasma TG levels dramatically in patients with no LpL suggested that hepatic uptake must have increased in association with reduced circulating apoCIII concentrations (10, 43). Interestingly, kinetic studies of VLDL apoB and TG metabolism in subjects heterozygous for LOF *APOC3* mutations demonstrated greater conversion of VLDL to LDL and no difference in hepatic uptake of VLDL in those individuals compared to their unaffected siblings (45).

At this time, the underlying mechanism causing thrombocytopenia during volanesorsen treatment has not been identified, although lowering of platelet counts seem to be dose-dependent (44). A GalNAc3 conjugated APOC3 ASO (developed by IONIS), which targets the ASO to the liver with very high efficiency, is currently being developed and appears, possibly because of similar efficacy with significantly reduced doses, to have a much improved safety profile (46).

### Targeting Angiopoietin-Like Proteins

Angiopoietin-like proteins (ANGPTL) are structurally similar to the angiopoietins, a family of proteins involved in angiogenesis (47). ANGPTL1 to ANGPTL7 each have an amino-terminal coiled-coil domain, a linker region, and a carboxy-terminal fibrinogen-like domain. ANGPTL8 contrasts with the other members of angiopoietin-like protein family because of it lacks a carboxy-terminal fibrinogen-like domain (FLD) (48). Below we describe studies exploring the lipid altering effects of 3 of the ANGPTL family members, ANGPTL3, 4, and 8, which are being developed to treat various forms of dyslipidemia.

#### Angiopoietin-Like Protein 3

ANGPTL3 is a secretory protein primarily expressed in the liver and encoded by a member of the angiopoietin-like family of genes. Angplt3 was identified initially as the cause of hypolipidemia in KK mice with a spontaneous mutation in the gene (49). ANGPTL3 inhibits LPL and endothelial lipase (EL) hydrolysis of triglycerides and phospholipids (49, 50). LOF mutations in *ANGPTL3* results in pan-hypolipidemia and the gene has been shown by Mendelian randomization studies to be causal for CVD (27, 51). These results have led to development of biologics, including ASO and monoclonal antibodies, that can reduce circulating levels of ANGPTL3.

ANGPTL3-LRx ASO (developed by IONIS) is taken up with high specificity by the liver where it targets *ANGPTL3* mRNA (52). Studies of gene deletion or mRNA knockdown in mice demonstrated reductions TG and LDL cholesterol levels, although effects on HDL cholesterol levels varied by the mouse model used. Effects of the ASO in mice led to reductions in plasma lipids as well as reductions in liver triglyceride content, atherosclerosis progression, and increases in insulin sensitivity. In the same publication, first-in-man studies showed that after 6 weeks of treatment, ANGPTL3-LRx ASO resulted in reductions in levels of ANGPTL3 protein (reductions of 46.6–84.5% from baseline depending on dose used) and in levels of triglycerides (reductions of 33.2–63.1%), LDL cholesterol (1.3–32.9%), very-low-density lipoprotein cholesterol (27.9–60.0%), non–high-density lipoprotein cholesterol (10.0–36.6%), apolipoprotein B (3.4–25.7%), and apolipoprotein C-III (18.9–58.8%). Three participants who received the antisense oligonucleotide and three who received placebo reported dizziness or headache. There were no serious adverse events (52).

Evinacumab is a monoclonal antibody (developed by Regeneron) that inhibits ANGPTL3. In two preclinical studies, inhibition of ANGPTL3 resulted in reduced secretion of VLDL TG by the liver and lower plasma lipid levels (53, 54). In randomized, double-blind, placebo-controlled, Phase 1 studies, evinacumab lowered both TG's and VLDL-C. In the single ascending dose study, the highest dose lowered TG levels by 80%, and this decline was more noticeable with intravenous (IV) doses rather than subcutaneous (SC) doses. In the multiple ascending dose study, TG levels were reduced 70% (55). An open-label study of Evinacumab involving nine adults with homozygous familial hypercholesterolemia delivered promising results; TG levels were reduced by 47% and LDL-C was reduced by 49% after 4 weeks of treatment. Apo B, non-HDL cholesterol and HDL decreased 46, 49, and 36%, respectively (56).

#### Angiopoietin-Like Protein 4

ANGPTL4 (angiopoietin-like protein 4/fasting-induced adipose factor) is an inhibitor of LpL that is expressed in many tissues and regulates lipid metabolism, especially TG levels (57, 58). As was the case for ANGPTL3, LOF mutations in ANGPTL4 were associated with lower TG levels and lower risk for CVD in a large cohort study (59). Unfortunately, knock out mice fed with a high fat diet had a high mortality rate and numerous lipo-granulomatous lesions in the intestines and mesenteric lymph nodes (58, 60). This curtailed targeting ANGPTL4 as a potential approach to treat dyslipidemia.

#### Angiopoietin-Like Protein 8

ANGPTL8 is expressed in liver and adipose tissue (61). It appears to interact with ANGPTL3 to increase TG levels, and a study in mice showed that ANGPTL8 does not inhibit LPL directly, but instead acts with ANGPTL3 or possibly other ANGPTL family members to increase TG levels. The possibility of inhibiting ANGPTL8 clinically is being considered.

### Treatments Targeting Apolipoprotein CII

ApoCII is the necessary activator of LPL and complete deficiency causes a phenotype that is identical to that of LpL deficiency (8). Although the severe HTG of apoCII deficiency can be treated by plasma transfusions (62), a practical chronic therapy for this very rare disorder is needed. Recent studies indicate that treatment with an apoCII mimetic peptide may alleviate the severe HTG in patients with complete loss of apoCII beyond standard management (13, 63).

### Treatments Targeting Lipoprotein Lipase Gene

Lipoprotein Lipase Deficiency (LPLD) is the most common of the very rare monogenic disorders that cause FCS. It typically presents in childhood with signs associated with severe HGT such as eruptive xanthomas, pancreatitis, and recurrent episodes of abdominal pain (8). Fibrates and omega-3-fatty acids are not sufficient to manage this condition and diet control, requiring no more than 10% of calories of fat, is very difficult in terms of compliance. Alipogene tiparvovec (marketed as Glybera) was approved in Europe in 2012. It was a hyperactive recombinant LpL with the p.S447X variant that was delivered via injection of an adeno-associated viral vector. Initial response was excellent, but TG levels reverted toward pretreatment levels after several months (64). Additionally, it was extremely expensive. Hence, in 2017, the sponsor declined to pursue a renewal for market authorization.

## Author Contributions

All authors listed have made a substantial, direct and intellectual contribution to the work, and approved it for publication.

## Conflict of Interest

The authors declare that the research was conducted in the absence of any commercial or financial relationships that could be construed as a potential conflict of interest. The handling Editor declared a past co-authorship with the author HG.

## References

[1] Choi SH Ginsberg HN. Increased very low density lipoprotein (VLDL) secretion, hepatic steatosis, and insulin resistance. Trends Endocrinol Metab. (2011) 22:353–63. 10.1016/j.tem.2011.04.007 21616678 PMC3163828

[2] Chapman MJ Ginsberg HN Amarenco P Andriotti F Boren J Catapano AL. Triglyceride-rich lipoproteins and high-density lipoprotein cholesterol in patients at high risk of cardiovascular disease: evidence and guidance for management. Eur Heart J. (2011) 32:1345–61. 10.1093/eurheartj/ehr112 21531743 PMC3105250

[3] Hegele RA Ginsberg HN Chapman MJ Nordestgaard BG Kuivenhoven JA Averna M. The polygenic nature of hypertriglyceridaemia: implications for definition, diagnosis, and management. Lancet Diabetes Endocrinol. (2014) 2:655–66. 10.1016/S2213-8587(13)70191-8 24731657 PMC4201123

[4] Packard CJ Boren J Taskinen M-R. Causes and consequences of hypertriglyceridemia. Front Endocrinol. (2020) 11:252. 10.3389/fendo.2020.00252 32477261 PMC7239992

[5] Adeli K Lewis GF. Intestinal lipoprotein overproduction in insulin-resistant states. Curr Opin Lipidol. (2008) 19:221–8. 10.1097/MOL.0b013e3282ffaf82 18460911

[6] Reaven GM Hill DB Gross RC Farquhar JW. Kinetics of triglyceride turnover of very low density lipoproteins of human plasma. J Clin Invest. (1965) 44:1826–33. 10.1172/JCI105290 5843713 PMC289683

[7] Borén J Watts GF Adiels M Söderlund S Chan DC Hakkarainen A. Kinetic and related determinants of plasma triglyceride concentration in abdominal obesity: multicenter tracer kinetic study. Arterioscler Thromb Vasc Biol. (2015) 35:2218–24. 10.1161/ATVBAHA.115.305614 26315407

[8] Hegele RA Borén J Ginsberg HN Arca M Averna M Binder CJ. Rare dyslipidaemias, from phenotype to genotype to management: a European Atherosclerosis Society task force consensus statement. Lancet Diabetes Endocrinol. (2020) 8:50–67. 10.1016/S2213-8587(19)30264-5 31582260

[9] Basu D Goldberg IJ. Regulation of lipoprotein lipase-mediated lipolysis of triglycerides. Curr Opin Lipidol. (2020) 31:154–60. 10.1097/MOL.0000000000000676 32332431 PMC7478854

[10] Gordts PL Nock R Son NH Ramms B Lew I Gonzales JC. ApoC-III inhibits clearance of triglyceride-rich lipoproteins through LDL family receptors. J Clin Invest. (2016) 126:2855–66. 10.1172/JCI86610 27400128 PMC4966320

[11] Chait A Ginsberg HN Vaisar T Heinecke JW Goldberg IJ Bornfeldt KE. Remnants of the triglyceride-rich lipoproteins, diabetes, and cardiovascular disease. Diabetes. (2020) 69:508–16. 10.2337/dbi19-0007 32198194 PMC7085249

[12] Brahm AJ Hegele RA. Chylomicronaemia–current diagnosis and future therapies. Nat Rev Endocrinol. (2015) 11:352–62. 10.1038/nrendo.2015.26 25732519

[13] Wolska A Reimund M Remaley AT. Apolipoprotein C-II: the re-emergence of a forgotten factor. Curr Opin Lipidol. (2020) 31:147–53. 10.1097/MOL.0000000000000680 32332429

[14] Young SG Fong LG Beigneux AP Allan CM He C Jiang H. GPIHBP1 and lipoprotein lipase, partners in plasma triglyceride metabolism. Cell Metab. (2019) 30:51–65. 10.1016/j.cmet.2019.05.023 31269429 PMC6662658

[15] Doolittle MH Ehrhardt N Péterfy M. Lipase maturation factor 1: structure and role in lipase folding and assembly. Curr Opin Lipidol. (2010) 21:198–203. 10.1097/MOL.0b013e32833854c0 20224398 PMC3924775

[16] Laufs U Parhofer KG Ginsberg HN Hegele RA. Clinical review on triglycerides. Eur Heart J. (2020) 41:99–109c. 10.1093/eurheartj/ehz785 31764986 PMC6938588

[17] Davidson M Stevenson M Hsieh A Ahmad Z Roeters van Lennep J. The burden of familial chylomicronemia syndrome: results from the global IN-FOCUS study. J Clin Lipidol. (2018) 12:898–907.e2. 10.1016/j.jacl.2018.04.009 29784572

[18] Havel RJ. Pathogenesis, differentiation and management of hypertriglyceridemia. Adv Intern Med. (1969) 15:117–54. 4908616

[19] Yang F Wang Y Sternfeld L Rodriguez JA Ross C Hayden MR. The role of free fatty acids, pancreatic lipase and Ca+ signalling in injury of isolated acinar cells and pancreatitis model in lipoprotein lipase-deficient mice. Acta Physiol. (2009) 195:13–28. 10.1111/j.1748-1716.2008.01933.x 18983441

[20] Vallejo-Vaz AJ Corral P Schreier L Ray KK. Triglycerides and residual risk. Curr Opin Endocrinol Diabetes Obes. (2020) 27:95–103. 10.1097/MED.0000000000000530 32073428

[21] Berglund L Brunzell JD Goldberg AC Goldberg IJ Sacks F Murad MH. Endocrine society. J Clin Endocrinol Metab. (2012) 97:2969–89. 10.1210/jc.2011-3213 22962670 PMC3431581

[22] Emerging Risk Factors Collaboration Di Angelantonio E Sarwar N Perry P Kaptoge S Ray KK. Major lipids, apolipoproteins, and risk of vascular disease. JAMA. (2009) 302:1993–2000. 10.1001/jama.2009.1619 19903920 PMC3284229

[23] Triglyceride Coronary Disease Genetics Consortium Emerging Risk Factors Collaboration Sarwar N Sandhu MS Butterworth AS Di Angelantonio E Boekholdt SM. Triglyceride-mediated pathways and coronary disease: collaborative analysis of 101 studies. Lancet. (2010) 375:1634–9. 10.1016/S0140-6736(10)60545-4 20452521 PMC2867029

[24] Pollin TI Damcott CM Shen H Ott SH Shelton J Horenstein RB. A null mutation in human APOC3 confers a favorable plasma lipid profile and apparent cardioprotection. Science. (2008) 322:1702–5. 10.1126/science.1161524 19074352 PMC2673993

[25] TG and HDL Working Group of the Exome Sequencing Project, National Heart, Lung, Blood Institute Crosby J Peloso GM. Loss-of-function mutations in APOC3, triglycerides, and coronary disease. N Engl J Med. (2014) 371:22–31. 10.1056/NEJMoa1307095 24941081 PMC4180269

[26] Khera AV Won HH Peloso GM O'Dushlaine C Liu D Stitziel NO. Association of rare and common variation in the lipoprotein lipase gene with coronary artery disease. JAMA. (2017) 317:937–46. 10.1001/jama.2017.0972 28267856 PMC5664181

[27] Stitziel NO Khera AV Wang X Bierhals AJ Vourakis AC Spery AE. ANGPTL3 deficiency and protection against coronary artery disease. J Am Coll Cardiol. (2017) 69:2054–63. 10.1016/j.jacc.2017.02.030 28385496 PMC5404817

[28] Mach F Baigent C Catapano AL Koskinas KC Casula M Badimon L. 2019 ESC/EAS Guidelines for the management of dyslipidaemias: lipid modification to reduce cardiovascular risk. Eur Heart J. (2020) 41:111–88. 10.1093/eurheartj/ehz455 31504418

[29] ACCORD Study Group Ginsberg HN Elam MB Lovato LC Crouse JR 3rd Leiter LA. Effects of combination lipid therapy in type 2 diabetes mellitus. N Engl J Med. (2010) 362:1563–74. 10.1056/NEJMoa1001282 20228404 PMC2879499

[30] Fruchart JC Santos RD Aguilar-Salinas C Aikawa M Al Rasadi K Amarenco P. The selective peroxisome proliferator-activated receptor alpha modulator (SPPARMα) paradigm: conceptual framework and therapeutic potential : A consensus statement from the International Atherosclerosis Society (IAS) and the Residual Risk Reduction Initiative (R3i) Foundation. Cardiovasc Diabetol. (2019) 18:71. 10.1186/s12933-019-0864-7 31164165 PMC6549355

[31] Ida S Kaneko R Murata K. Efficacy and safety of pemafibrate administration in patients with dyslipidemia: a systematic review and meta-analysis. Cardiovasc Diabetol. (2019) 18:38. 10.1186/s12933-019-0845-x 30898163 PMC6429757

[32] Pradhan AD Paynter NP Everett BM Glynn RJ Amarenco P Elam M. Rationale and design of the pemafibrate to reduce cardiovascular outcomes by reducing triglycerides in patients with diabetes (PROMINENT) study. Am Heart J. (2018) 206:80–93. 10.1016/j.ahj.2018.09.011 30342298

[33] Swanson D Block R Mousa SA. Omega-3 fatty acids EPA and DHA: health benefits throughout life. Adv Nutr. (2012) 3:1–7. 10.3945/an.111.000893 22332096 PMC3262608

[34] GISSI-Prevenzione Investigators Dietary supplementation with n-3 polyunsaturated fatty acids and vitamin E after myocardial infarction: results of the GISSI-Prevenzione trial. Lancet. (1999) 354:447–55. 10.1016/S0140-6736(99)07072-5 10465168

[35] Aung T Halsey J Kromhout D Gerstein HC Marchioli R Tavazzi L. Associations of omega-3 fatty acid supplement use with cardiovascular disease risks: meta-analysis of 10 trials involving 77 917 individuals. JAMA Cardiol. (2018) 3:225–34. 10.1001/jamacardio.2017.5205 29387889 PMC5885893

[36] ASCEND Study Collaborative Group. Bowman L Mafham M Wallendszus K Stevens W Buck G Barton J. Effects of n-3 fatty acid supplements in diabetes mellitus. N Engl J Med. (2018) 379:1540–50. 10.1056/NEJMoa1804989 30146932

[37] Manson JE Cook NR Lee IM Christen W Bassuk SS Mora S. Marine n-3 fatty acids and prevention of cardiovascular disease and cancer. N Engl J Med. (2019) 380:23–32. 10.1056/NEJMoa1811403 30415637 PMC6392053

[38] Yokoyama M Origasa H Matsuzaki M Matsuzawa Y Saito Y Ishikawa Y. Effects of eicosapentaenoic acid on major coronary events in hypercholesterolaemic patients (JELIS): a randomised open-label, blinded endpoint analysis [published correction appears in Lancet. Lancet. (2007) 369:1090–8. 10.1016/S0140-6736(07)60527-3 17398308

[39] Bhatt DL Steg G Miller M Matsuzawa Y Ishikawa Y Oikawa S. For the REDUCE-IT Investigators. Cardiovascular risk reduction with icosapent ethyl for hypertriglyceridemia. N Engl J Med. (2019) 380:11–22. 10.1056/NEJMoa1812792 30415628

[40] Hu Y Hu FB Manson JE. Marine omega-3 supplementation and cardiovascular disease: an updated meta-analysis of 13 randomized controlled trials involving 127 477 participants. J Am Heart Assoc. (2019) 8:e013543. 10.1161/JAHA.119.013543 31567003 PMC6806028

[41] Reyes-Soffer G Ginsberg HN. Life is complicated: so is apoCIII. J Lipid Res. (2019) 60:1347–9. 10.1194/jlr.C119000214 31239286 PMC6672043

[42] Jørgensen AB Frikke-Schmidt R Nordestgaard BG Tybjærg-Hansen A. Loss-of-function mutations in APOC3 and risk of ischemic vascular disease. N Engl J Med. (2014) 371:32–41. 10.1056/NEJMoa1308027 24941082

[43] Gaudet D Brisson D Tremblay K Alexander VJ Singleton W Hughes SG. Targeting APOC3 in the familial chylomicronemia syndrome. N Engl J Med. (2014) 371:2200–6. 10.1056/NEJMoa1400284 25470695

[44] Witztum JL Gaudet D Freedman SD Alexander VJ Digenio A Williams KR. Volanesorsen and triglyceride levels in familial chylomicronemia syndrome. N Engl J Med. (2019) 381:531–42. 10.1056/NEJMoa1715944 31390500

[45] Reyes-Soffer G Sztalryd C Horenstein RB Holleran S Matveyenko A Thomas T. Effects of APOC3 heterozygous deficiency on plasma lipid and lipoprotein metabolism. Arterioscler Thromb Vasc Biol. (2019) 39:63–72. 10.1161/ATVBAHA.118.311476 30580564 PMC6309928

[46] Alexander VJ Xia S Hurh E Hughes S O'Dea L Geary RS. N-acetyl galactosamine-conjugated antisense drug to APOC3 mRNA, triglycerides and atherogenic lipoprotein levels. Eur Heart J. (2019) 40:2785–96. 10.1093/eurheartj/ehz209 31329855 PMC6736334

[47] Santulli G. Angiopoietin-like proteins: a comprehensive look. Front Endocrinol. (2014) 5:4. 10.3389/fendo.2014.00004 24478758 PMC3899539

[48] Quagliarini F Wang Y Kozlitina J Grishin NV Hyde R Boerwinkle E. Atypical angiopoietin-like protein that regulates ANGPTL3. Proc Natl Acad Sci USA. (2012) 109:19751–6. 10.1073/pnas.1217552109 23150577 PMC3511699

[49] Koishi R Ando Y Ono M Shimamura M Yasumo H Fujiwara T. Angptl3 regulates lipid metabolism in mice. Nat Genet. (2002) 30:151–7. 10.1038/ng814 11788823

[50] Arca M D'Erasmo L Minicocci I. Familial combined hypolipidemia: angiopoietin-like protein-3 deficiency. Curr Opin Lipidol. (2020) 31:41–8. 10.1097/MOL.0000000000000668 32022755

[51] Dewey FE Gusarova V Dunbar RL O'Dushlaine C Schurmann C Gottesman O. Genetic and pharmacologic inactivation of ANGPTL3 and cardiovascular disease. N Engl J Med. (2017) 377:211–21. 10.1056/NEJMoa1612790 28538136 PMC5800308

[52] Graham MJ Lee RG Brandt TA Tai L-J Fu W Peralta R. Cardiovascular and metabolic effects of ANGPTL3 antisense oligonucleotides. N Engl J Med. (2017) 377:222–32. 10.1056/NEJMoa1701329 28538111

[53] Gusarova V Alexa CA Wang Y Rafique A Kim JH Buckler D. ANGPTL3 blockade with a human monoclonal antibody reduces plasma lipids in dyslipidemic mice and monkeys. J Lipid Res. (2015) 56:1308–17. 10.1194/jlr.M054890 25964512 PMC4479335

[54] Wang Y Gusarova V Banfi S Gromada J Cohen JC Hobbs HH. Inactivation of ANGPTL3 reduces hepatic VLDL-triglyceride secretion. J Lipid Res. (2015) 56:1296–307. 10.1194/jlr.M054882 25954050 PMC4479334

[55] Ahmad Z Banerjee P Hamon S Chan K-C Bouzelmat A Sasiela WJ. Inhibition of angiopoietin-like protein 3 with a monoclonal antibody reduces triglycerides in hypertriglyceridemia. Circulation. (2019) 140:470–86. 10.1161/CIRCULATIONAHA.118.039107 31242752 PMC6686956

[56] Gaudet D Gipe DA Pordy R Ahmad Z Cuchel M Shah PK. ANGPTL3 inhibition in homozygous familial hypercholesterolemia. N Engl J Med. (2017) 377:296–7. 10.1056/NEJMc1705994 28723334

[57] Yoshida K Shimizugawa T Ono M Furukawa H. Angiopoietin-like protein 4 is a potent hyperlipidemia-inducing factor in mice and inhibitor of lipoprotein lipase. J Lipid Res. (2002) 43:1770–2. 10.1194/jlr.C200010-JLR200 12401877

[58] Desai U Lee EC Chung K Gao C Gay J Key B. Lipid-lowering effects of anti-angiopoietin-like 4 antibody recapitulate the lipid phenotype found in angiopoietin-like 4 knockout mice. Proc Natl Acad Sci USA. (2007) 104:11766–71. 10.1073/pnas.0705041104 17609370 PMC1913890

[59] Dewey FE Gusarova V O'Dushlaine C Gottesman O Trejos J Hunt C. Inactivating variants in ANGPTL4 and risk of coronary artery disease. N Engl J Med. (2016) 374:1123–33. 10.1056/NEJMoa1510926 26933753 PMC4900689

[60] Lichtenstein L Mattijssen F de Wit NJ Georgiadi A Hooiveld GJ van der Meer R. Angptl4 protects against severe proinflammatory effects of saturated fat by inhibiting fatty acid uptake into mesenteric lymph node macrophages. Cell Metab. (2010) 12:580–92. 10.1016/j.cmet.2010.11.002 21109191 PMC3387545

[61] Haller JF Mintah IJ Shihanian LM Stevis P Buckler D Alexa-Braun CA. ANGPTL8 requires ANGPTL3 to inhibit lipoprotein lipase and plasma triglyceride clearance. J Lipid Res. (2017) 58:1166–73. 10.1194/jlr.M075689 28413163 PMC5454515

[62] Breckenridge WC Little JA Steiner G Chow A Poapst M. Hypertriglyceridemia associated with deficiency of apolipoprotein C-II. N Engl J Med. (1978) 298:1265–73. 10.1056/NEJM197806082982301 565877

[63] Wolska A Lo L Sviridov DO Pourmousa M Pryor M Ghosh SS. A dual apolipoprotein C-II mimetic-apolipoprotein C-III antagonist peptide lowers plasma triglycerides. Sci Transl Med. (2020) 12:eaaw7905. 10.1126/scitranslmed.aaw7905 31996466 PMC8359806

[64] Gaudet D Méthot J Déry S Brisson D Essiembre C Tremblay G. Efficacy and long-term safety of alipogene tiparvovec (AAV1-LPLS447X) gene therapy for lipoprotein lipase deficiency: an open-label trial. Gene Ther. (2013) 20:361–9. 10.1038/gt.2012.43 22717743 PMC4956470

